# Analysis of LIN28A in early human ovary development and as a candidate gene for primary ovarian insufficiency

**DOI:** 10.1016/j.mce.2011.12.016

**Published:** 2012-04-04

**Authors:** Ranna El-Khairi, Rahul Parnaik, Andrew J. Duncan, Lin Lin, Dianne Gerrelli, Mehul T. Dattani, Gerard S. Conway, John C. Achermann

**Affiliations:** a Developmental Endocrinology Research Group, Clinical and Molecular Genetics Unit, University College London (UCL) Institute of Child Health, University College London, London WC1N 1EH, United Kingdom; b Human Developmental Biology Resource, Neural Development Unit, University College London (UCL) Institute of Child Health, University College London, London WC1N 1EH, United Kingdom; c Department of Endocrinology, University College London Hospitals, London NW1 2PQ, United Kingdom

**Keywords:** CS, carnegie stage, FSH, follicle-stimulating hormone, DAPI, 4′,6-diamidino-2-phenylindol, GAPDH, glyceraldehyde-3-phosphate dehydrogenase, HDBR, human developmental biology resource, miRNA, microRNA, PBS, phosphate-buffered saline, PGC, primordial germ cell, POI, primary ovarian insufficiency, qRT-PCR, quantitative real-time PCR, TBST, Tris-buffered saline with 1% Tween20, wpc, weeks post conception, Lin28A, Lin28B, Primary ovarian insufficiency (POI), Premature ovarian failure (POF), Germ cell

## Abstract

Lin28 proteins are emerging as important regulators of microRNAs in endocrine systems. *Lin28a* regulates primordial germ cell development and puberty timing in mice, whereas the related protein *LIN28B* is associated with age at menarche in genome-wide association studies in humans. Here, we studied expression of *LIN28A* and *LIN28B* in early human gonad development. *LIN28A* increased in the developing ovary between 6 and 9 weeks post conception, but not in the developing testis. Immunohistochemistry demonstrated LIN28A in peripheral germ cells. *LIN28B* was expressed at lower levels in both tissues and did not increase with time. As disruption of *Lin28a* affects germ cell development in mice, *LIN28A* was considered a candidate gene for primary ovarian insufficiency (POI) in humans. However, no significant changes were found in 50 women studied. These findings show LIN28A is strongly expressed in germ cells during early human ovary development, but disruption of *LIN28A* is not a common cause of POI.

## Highlights

► Lin28 proteins regulate micro RNAs and are implicated in germ cell development. ► We assessed *LIN28A* and *LIN28B* in the early human ovary and testis. ► Expression of *LIN28A* increases in the developing ovary between 6 and 9 weeks conception. ► Expression of *LIN28B* was lower and did not change. ► Alterations in *LIN28A* were not found in women with primary ovarian insufficiency.

## 1 Introduction

LIN28A and LIN28B are highly conserved RNA-binding proteins that block biogenesis of the microRNA (miRNA), *let-7*, thereby promoting stem/progenitor cell proliferation and inhibiting differentiation (Viswanathan et al., 2008; Viswanathan and Daley, 2010). These factors are emerging as important regulators of several endocrine systems. *Lin28a* (also known as *Lin28*) regulates primordial germ cell development in mice. Deletion of this gene results in a reduced germ cell pool in mouse embryos, whereas overexpression of *Lin28a* results in greater germ cell number and larger first litter size (West et al., 2009; Zhu et al., 2010). Alterations in this gene also affect body size, insulin sensitivity and timing of puberty (Zhu et al., 2010). The related factor, *LIN28B* has also been implicated in growth and puberty as polymorphic variability in *LIN28B* is strongly associated with age at menarche and stature in several independent genome-wide association studies in humans (He et al., 2009, 2010; Ong et al., 2009; Perry et al., 2009; Sulem et al., 2009; Widen et al., 2010).

Despite these recent advances, relatively little is known about the role of the LIN28 family of proteins in the developing human female reproductive system, or in clinical reproductive disorders. We hypothesized that LIN28A and LIN28B could have an important role in human germ cell development and expansion of the germ cell pool, and that disruption of these genes might lead to germ cell depletion and primary ovarian insufficiency (POI). To address this, a study was undertaken to investigate expression of *LIN28A* and *LIN28B* in early human ovary development and, based on our findings, mutational analysis of *LIN28A* was undertaken in a cohort of women with POI.

## 2 Materials and methods

### 2.1 RNA preparation

Human testes and ovaries (*n* = 3 each) from Carnegie stage (CS) 18–22 (6–7 weeks post conception, wpc), Fetal stage 1 (F1) (8 wpc) and F2 (9 wpc) were obtained from the Medical Research Council/Wellcome Trust funded Human Developmental Biology Resource (HDBR, www.hdbr.org), with Research Ethics Committee approval and informed consent. Samples were obtained on ice and preserved in RNA later (Ambion, Austin, TX, USA). Fetal heart tissue (8 wpc) was used as a control. RNA was extracted using the Trizol method and the concentration and ratio of absorbance at 260–280 nm (A260/A280 ratio) were measured using a NanoDrop ND-1000 Spectrophotometer (NanoDrop Technologies, Witec, Littau, Switzerland). First-strand cDNA was synthesized using the SuperScript II Reverse Transcriptase (Invitrogen, Paisley, UK) and random primers according to the manufacturer’s instructions. The amount of input RNA in each reaction was calculated to be 200 ng.

### 2.2 qRT-PCR of LIN28A and LIN28B

Primers and TaqMan® probes were obtained for *LIN28A* and *LIN28B* (Applied Biosystems, Warrington, UK). Amplification was performed in a total volume of 20 μl per reaction using TaqMan® Gene Expression Master Mix and a StepOnePlus™ Real-time PCR System (Applied Biosystems). Thermocycling conditions consisted of an initial step of 2 min at 50 °C, denaturation of 10 min at 95 °C, followed by 40 cycles of 95 °C for 15 s and 60 °C for 1 min. Human glyceraldehyde-3-phosphate dehydrogenase (*GAPDH*; 4333764T) was used for normalization and relative quantification of gene expression was performed according to the 2^−ΔΔCt^ method (Livak and Schmittgen, 2001). Data were analyzed with StepOne software version 2.1 and results expressed as fold change above control.

### 2.3 Immunohistochemistry of LIN28A

Ovaries from a CS22 embryo (7 wpc) or testis from a F2 fetus (9 wpc) were obtained from the Human Developmental Biology Resource, sunk and positioned in Tissue-Tek® O.C.T. (Fisher Scientific, Loughborough, UK) and frozen on dry ice. Frozen sections were cut at 12–14 microns thickness, collected on Superfrost slides (Fisher Scientific), dried and stored at −20 °C. For immunohistochemistry, sections were fixed for three minutes by immersion in 4% paraformaldehyde in phosphate-buffered saline (PBS), rinsed immediately in Tris-buffered saline with 1% Tween 20 (TBST) or PBS, and incubated for 1 h in blocking buffer (10% Lamb Serum or 1% BSA in TBST). Sections were incubated overnight at 4 °C using the following primary antibodies: mouse anti-LIN28A monoclonal (1:200 dilution) (New England Biolabs, Hitchin, UK; 5930S); rabbit anti-POU5F1 (OCT4) polyclonal (1:200) (Abcam, Cambridge, UK; Ab19857). Slides were washed in TBST, before incubation overnight in 4′,6-diamidino-2-phenylindol (DAPI) (10 μg/ml) and the following secondary antibodies: goat anti-mouse polyclonal, conjugated to Alexa555 (1:400) (Invitrogen, A11001); goat anti-rabbit polyclonal, conjugated to Alexa488 (1:400) (Invitrogen, A21429). Slides were washed and mounted in Vectashield (Vectorlabs, Peterborough, UK) then visualized on an inverted Zeiss LSM 710 confocal microscope (Carl Zeiss Ltd., Welwyn Garden City, UK). Controls were obtained following omission of the primary antibodies.

### 2.4 Sequence analysis of LIN28A

After institutional board approval and with consent, direct sequencing of the entire coding region of *LIN28A* was undertaken in a cohort of 50 patients with POI of unknown etiology. POI was defined as amenorrhea of at least six months duration presenting before the age of 40 years with a serum follicle-stimulating hormone (FSH) measurement of greater than 20 IU/l on two occasions. This cohort included 13 patients with POI and primary amenorrhea and 37 women with POI and secondary amenorrhea (mean age of onset, 24.7 years). A family history of POI was present in 13 cases. Women with Fragile X premutations were excluded and analysis of the FSH receptor was negative.

Primers were designed using Primer3 online software (version 0.4.0, http://frodo.wi.mit.edu/primer3/) for the four coding exons of *LIN28A* (Ensembl transcript ENST00000326279) (Table 1). PCR reactions were performed using MegaMix (Microzone Ltd., Haywards Heath, UK) and 0.5 μl of forward and reverse primers (5–25 μM, Eurofins MWG Operon, Germany) in 20 μl reaction volume, for 35 cycles and with an annealing temperature of 55 °C. Amplicons were purified using Microclean (Microzone Ltd.) and then subjected to direct sequencing using dye terminator sequencing kits (BigDye® Terminator v1.1, Applied Biosystems, Warrington, UK) using an automated capillary based sequencer (Applied Biosystems). Sequencher version 4.6 (Genecodes Corp., Ann Arbor, MI) was used to analyze the data.

## 3 Results

### 3.1 LIN28A expression increases at key stages of early human ovary development

**Fig. 1:**
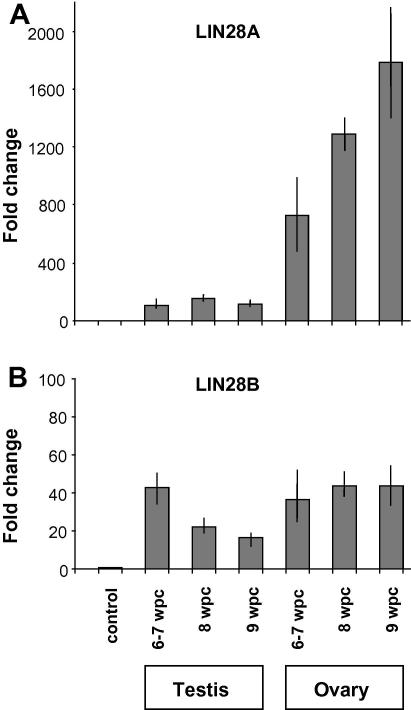
Expression of *LIN28A* and *LIN28B* in the gonad between 6 and 9 weeks post conception (wpc). (A) Ovary and testis samples showed higher expression of *LIN28A* compared to control tissue (heart; 8wpc). Similar results were seen when other control samples were studied (data not shown). A progressive increase in *LIN28A* expression was detected in the ovary up until 9 wpc. (B) *LIN28B* expression was higher in the testis and ovary compared to control, but did not show a marked increase with ovarian age. Data are shown as fold change above control, with *LIN28A* and *LIN28B* expression levels normalized to GAPDH and relative quantification of gene expression performed according to the 2^−ΔΔCt^ method. Bars represent 1SD. Note the different scales in panels A and B.

Analysis of *LIN28A* and *LIN28B* in human fetal gonads between 6 and 7 wpc showed significantly higher expression of *LIN28A* in the developing ovary compared to testis and to control tissue (heart) (Fig. 1A). A further increase in *LIN28A* transcript levels was seen with increasing age in the ovary whereas expression levels in the testis remained constant (Fig. 1A). Differential expression of *LIN28B* was also higher in the gonads compared to control tissue (heart), but did not show marked differences between the ovary and testis (Fig. 1B).

### 3.2 Immunohistochemistry of LIN28A

**Fig. 2:**
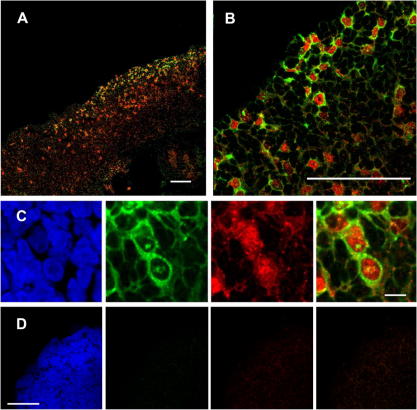
Immunohistochemistry showing expression of POU5F1 (OCT4) (red) and LIN28A (green) in the human ovary at 7 wpc. (A) Low power image showing a population of LIN28A expressing cells in the peripheral cortical region of the developing gland. Lower level expression of LIN28A was seen in some somatic cells. Scale bar, 100 μm. (B) LIN28A shows strong staining in this population of POU5F1 (OCT4)-expressing germ cells. Scale bar, 100 μm. (C) High power image showing LIN28A staining mainly in the cytoplasm of germ cells whereas POU5F1 (OCT4) staining was intense in the nuclei. Scale bar, 10 μm. (D) The omission of primary antibody resulted in very minimal signal. Scale bar, 100 μm. Nuclei are counterstained with DAPI (blue).

Immunohistochemistry revealed strong expression of LIN28A together with POU5F1 (OCT4) in a population of germ cells in the developing human ovary at 7 wpc (Fig. 2A). LIN28A staining was strongest in a population of germ cells at the peripheral cortical region of the developing gland (Fig. 2B). Consistent with previous studies in mice, POU5F1 (OCT4) staining was confined to the nucleus of these cells whereas LIN28A staining was found to localize predominantly in the cytoplasm (Fig. 2C) (West et al., 2009). LIN28A staining was also seen in the nucleoli of several cells, and low levels of LIN28A expression was observed in somatic cells. No significant signal was seen in controls.

**Fig. 3:**
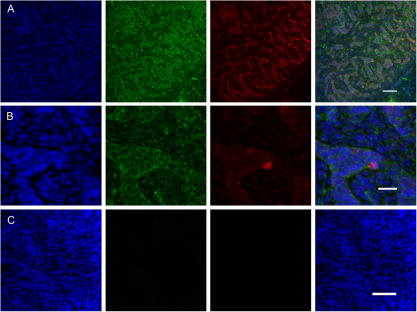
Immunohistochemistry showing expression of POU5F1 (OCT4) (red) and LIN28A (green) in the human testis at 9 wpc. (A) Low power image showing generalized low level expression of LIN28A with denser signal in the developing seminiferous cords. Scale bar, 100 μm. (B) High power image showing no clear increased intensity of LIN28A in POU5F1 (OCT4) expressing cells. Scale bar, 25 μm. (C) Control image showing omission of primary antibody resulted in no significant signal. Scale bar, 100 μm. Nuclei are counterstained with DAPI (blue).

Immunohistochemistry of human fetal testis at 9 wpc showed generalized low level expression of LIN28A with denser signal in the developing seminiferous cords (Fig. 3A). Stronger expression of LIN28A was generally not seen in POU5F1 (OCT4) positive cells (Fig. 3B).

### 3.3 Sequence analysis

We hypothesized that LIN28A could regulate primordial germ cell formation and expansion of the germ cell pool, and that disruption of *LIN28A* might lead to germ cell depletion and primary ovarian insufficiency (POI) in humans. Mutational analysis of the coding regions of *LIN28A* was therefore undertaken in a cohort of 50 women with POI of unknown etiology. However, no significant non-synonymous changes in the *LIN28A* gene were found in this cohort of women with POI.

## 4 Discussion

Primordial germ cells (PGCs) arise from pluripotent epiblast cells, and proliferate and migrate into the primitive gonad in all animal species. In humans, this process is thought to occur at approximately 4–5 weeks post-conception (wpc). In the ovary, germ cells (oogonia) undergo mitotic proliferation and a dramatic expansion in numbers. Many of these cells enter the prophase of meiosis and, following the S phase and almost the entire G2 phase (during which crossing-over occurs), demonstrate meiotic arrest at between 8 and 36 weeks (Baker, 1963; Kerr et al., 2008; Le et al., 2010; Skrzypczak et al., 1981). As a result of the mitotic proliferation a total of around 7 million germ cells are estimated to be present by 20 wpc. Many of these cells undergo apoptosis so that around 1 million germ cells are present at the time of birth and around 400 are ovulated during a woman’s reproductive lifespan (Broekmans et al., 2009). Germ cell formation and migration is essential for the development and maintenance of follicles in the ovary and for the organization and maintenance of ovarian structure. The establishment of primordial follicles early in ovarian development and their subsequent development into primary follicles has a direct consequence on the number of available oocytes throughout the female reproductive years (Edson et al., 2009).

Primary ovarian insufficiency (POI) is a heterogeneous condition which affects approximately 1% of women before the age of 40 years (Nelson, 2009). Although several single gene disorders associated with POI have been described, the underlying mechanisms responsible are likely to be diverse and in most cases are currently unknown. Studies from mouse models have shown that defects in PGC migration or proliferation (e.g. Steel, c-kit, Cxcr4) can be associated with subsequent ovarian germ cell depletion and ovarian insufficiency in postnatal or adult life (Edson et al., 2009; Jagarlamudi et al., 2010). Fewer data are available for humans, although it has been proposed that dysregulation of PGC development may be a mechanism for POI in some individuals.

In order to identify novel genes involved in POI we looked at the expression of *LIN28A* and *LIN28B* in the early human ovary. Lin28a and Lin28b selectively inhibit the processing of the *let-7* family of miRNA precursors into mature miRNAs (Viswanathan et al., 2008). LIN28A is emerging as a pluripotency factor and developmental regulator that may play an important role in promoting progenitor cell proliferation and in regulating germ cell differentiation in mice (Viswanathan and Daley, 2010; West et al., 2009; Zhu et al., 2010). LIN28A is also emerging as an important factor in oncogenesis and a marker of germ cell tumors in humans (Gillis et al., 2011; Viswanathan et al., 2009; West et al., 2009). Overexpression of LIN28A in these cells may suppress *let-7* processing, resulting in uncontrolled cell proliferation in the absence of controlled differentiation.

Our expression data show that *LIN28A* rather than *LIN28B* predominates during early human germ cell development. A progressive increase in *LIN28A* expression was measured by qRT-PCR in the developing ovary between 6 and 9 wpc. This finding may represent an increase in the LIN28A-positive population of germ cells during this critical early stage of development compared to the testis or control tissue. Furthermore, immunohistochemistry at 7 wpc revealed strong expression of LIN28A in a population of peripheral germ cells in the developing human ovary. These cells co-expressed nuclear POU5F1 (OCT4) (21) and represent a pool of developing germ cells. Weaker expression of LIN28A was seen in the testis, consistent with qRT-PCR findings. No strong co-localization with POU5F1 (OCT4) was seen.

Given these findings, and the data from *Lin28a* deleted mice, we hypothesized that disruption of *LIN28A* might lead to a failure of germ cell expansion and, ultimately, germ cell depletion and POI. However, we did not find any non-synonymous variants in *LIN28A* in a cohort of women with POI of unknown etiology. Further studies would be needed to establish whether disruption of LIN28A can be associated with more significant clinical phenotypes in rare cases, such as growth restriction and impaired insulin sensitivity, as well as ovarian insufficiency, or whether relative expression of LIN28A through copy number changes or through variable gene regulation could influence POI phenotypes. However, changes in the *LIN28A* gene are unlikely to be a common cause of more typical forms of POI.

In conclusion, this study shows that LIN28A is strongly expressed during early human germ cell development but that alterations in this gene are not a common cause of POI.

## 5 Disclosure summary

The authors have nothing to disclose. JCA holds a Wellcome Trust Senior Research Fellowship in Clinical Science (079666). The funding sources had no involvement in the conduct of the research, writing of the report, nor decision to submit the manuscript for publication.

## Figures and Tables

**Table 1:** Primers used for DNA amplification and sequencing of the four exons of *LIN28A*.

Exon	Primer name	Primer sequence (5′→3′)	PCR product size (bp)	Annealing temp (°C)
1	1F	tgggtcattgtcttttagaatttgg	295	55
	1R	cctaggactcagttccccacctc		
2	2F	tgtccacttgtggggctgga	400	55
	2R	cccgatggccctcaattcaac		
3	3F	cacatttgattcctaccctacagga	388	55
	3R	gggaccacaggcttcccttt		
4	4F	cagagcccaggtgtcctcattcg	400	55
	4R	tgccaactagccccaatgcac		

F: Forward primer; R: Reverse primer; bp: base pairs
